# Facial expression analysis to uncover the relationship between sialorrhea and hypomimia in Parkinson’s disease

**DOI:** 10.3389/fneur.2025.1661043

**Published:** 2025-10-24

**Authors:** Eline Serbée, Kye Won Park, Atefeh Irani, Maryam S. Mirian, Juana Ayala Castaneda, Michael Grundy, Martin J. McKeown

**Affiliations:** ^1^Pacific Parkinson’s Research Centre, University of British Columbia, Vancouver, BC, Canada; ^2^Division of Neurology, Department of Medicine, University of British Columbia, Vancouver, BC, Canada; ^3^Faculty of Medicine, Radboud University, Nijmegen, Netherlands; ^4^Department of Neurology, Gangneung Asan Hospital, University of Ulsan College of Medicine, Gangneung, Republic of Korea; ^5^School of Electrical and Computer Engineering, University of Tehran, Tehran, Iran

**Keywords:** Parkinson’s disease, drooling, sialorrhea, hypomimia, artificial intelligence

## Abstract

Sialorrhea, or excessive drooling, is a prevalent yet frequently under-recognized non-motor symptom of Parkinson’s disease (PD). Hypomimia, or reduced facial expressivity, constitutes another significant feature of PD. Although previous studies have suggested a potential clinical association between these two disease features, this relationship has seldom been quantified using artificial intelligence (AI) methodologies. In this study, we sought to characterize the association between hypomimia and sialorrhea in PD using both traditional clinical scales and AI-based video analysis. We conducted a cross-sectional study involving 52 individuals diagnosed with PD. Sialorrhea severity was assessed using the Radboud Oral Motor Inventory for Parkinson’s Disease–Saliva subscale (ROMP-saliva), while hypomimia was evaluated via the Unified Parkinson’s Disease Rating Scale (UPDRS). Facial video recordings were acquired and analyzed using AI algorithms to extract key facial landmarks. These landmarks were processed into 20 quantitative features representing the mouth, eyes, and combined facial regions. To assess the relationship between facial expressivity and sialorrhea severity, we employed Principal Component Analysis, Canonical Correlation Analysis, and bootstrapping. Clinical rating scales demonstrated a modest correlation between hypomimia and drooling severity (*r* = 0.368, *p* = 0.007). In contrast, video analysis revealed moderate correlations between ROMP-saliva scores and features derived from the mouth (mean *r* = 0.600), eyes (mean *r* = 0.641), and combined facial regions (mean *r* = 0.575). These findings support a quantifiable association between hypomimia and sialorrhea in PD and underscore the utility of quantitative facial analysis for the automated detection of under-recognized non-motor symptoms such as drooling.

## 1 Introduction

Parkinson’s disease (PD) is one of the fastest-growing neurodegenerative disorders, characterized by a constellation of motor and non-motor symptoms (1). The cardinal motor features of PD include bradykinesia, rigidity, and resting tremor. Hypomimia, or reduced facial expressivity, constitutes another significant feature of PD. Given that facial expression involves not merely facial muscular movements but also emotional and motivational aspects, hypomimia represents a symptom bridging both motor and non-motor domains (2). In addition, a wide range of non-motor symptoms—including gastrointestinal, genitourinary, sleep, and cognitive disturbances—are increasingly recognized as central characteristics of PD (3).

Sialorrhea, or excessive drooling, is a common non-motor manifestation in PD, affecting approximately 50% of patients, typically in the later stages of the disease (4, 5). The etiology of sialorrhea in PD is multifactorial, likely involving impaired oral motor control and diminished swallowing frequency, rather than hypersecretion of saliva (4). Clinically, sialorrhea is associated with adverse outcomes such as poor oral hygiene, increased risk of aspiration pneumonia, and significant psychosocial burden, such as that caused by embarrassment and social withdrawal (6). Despite its detrimental impact on quality of life, sialorrhea often goes unrecognized or unreported, partly due to its subtle presentation and the reluctance of patients to discuss it with healthcare providers. One survey revealed that fewer than half of affected individuals had ever discussed the symptom with their clinical team (6), highlighting a need for improved recognition and proactive screening.

Like sialorrhea, hypomimia represents a multifaceted symptom with both motor components (impaired facial muscle control and coordination) and potential non-motor elements (altered emotional expression and social communication). However, while sialorrhea typically remains hidden until patients voluntarily disclose it, facial expressions are externally visible and can be readily observed in the clinic (7). Emerging evidence suggests a potential association between hypomimia and sialorrhea in PD (4, 8). This relationship raises the possibility that facial expressivity—or its absence—may serve as a surrogate marker for detecting drooling. However, objective and validated tools to assess these symptoms, particularly sialorrhea, remain underutilized in clinical and research contexts. While semi-quantitative scales such as the Movement Disorder Society–sponsored Unified Parkinson’s Disease Rating Scale (MDS-UPDRS) are routinely used to assess hypomimia, these measures lack the granularity required for automated analyses. For sialorrhea assessment, the Radboud Oral Motor Inventory for Parkinson’s Disease–Saliva subscale (ROMP-saliva) represents the most validated instrument available, yet it entails similar limitations (9).

Technological advances in computer vision and artificial intelligence (AI) have enabled more objective and quantitative assessment of facial movement in PD (10). Automated video-based analysis can provide more precise and reproducible measurements of facial movements compared to conventional clinical scale-based methods (7). Given the reported link between hypomimia and sialorrhea, objective facial movement quantification may serve as a useful tool to indirectly detect drooling and enhance awareness of this symptom.

With such background, the present study aimed to (i) examine the association between hypomimia and sialorrhea using validated clinical scales, and (ii) further characterize their relationship through a quantitative facial video analysis.

## 2 Methods

### 2.1 Study population

A total of 104 individuals with PD were consecutively recruited between April 2023 and April 2024 from the Movement Disorders Clinic at the Djavad Mowafaghian Centre for Brain Health, University of British Columbia, Vancouver, Canada. Inclusion criteria comprised a clinical diagnosis of PD made by a movement disorder specialist in accordance with the United Kingdom Parkinson’s Disease Society Brain Bank criteria and an age of 18 years or older. Exclusion criteria included significant cognitive impairment precluding the completion of video-based tasks or questionnaires; the presence of identifiable medical or surgical conditions associated with dysautonomia; and any prior history of disorders affecting orofacial anatomy or salivary secretory function. The study was approved by the University of British Columbia Clinical Research Ethics Board, and all participants provided written, informed consent prior to enrollment.

### 2.2 Data and video collection

Participants completed the ROMP-saliva questionnaire to assess the prevalence and severity of drooling (9). For group-level comparisons, individuals with ROMP-saliva scores of 9 or below were classified as the non-drooling group, whereas scores greater than 9 were categorized as the drooling group. On the same day, facial expression videos were recorded using a dedicated camera module (OAK-D 12MP, Luxonis, Littleton, CO, United States) mounted above a desktop computer (iMac 2021, 24″, Apple Inc., Cupertino, CA, United States). Of note, this camera system was utilized as it was already installed for ongoing research projects. However, the facial landmark detection and subsequent analyses described in this study are applicable with standard webcam-quality video settings. Each participant recorded 10-s videos of emotional expressions with happiness being the emotion examined in this study. Specifically, participants were instructed to begin with a neutral face, transition to the target emotional expression, and return to a neutral expression by the end of the recording. Hypomimia was evaluated using item 3.2 of the Movement Disorder Society–sponsored Unified Parkinson’s Disease Rating Scale (MDS-UPDRS) (11) rated by a movement disorder specialist during the video recording session, blinded to the ROMP-saliva scores.

### 2.3 Data analysis

Video recordings were processed as follows. An automated emotion recognition module was employed to identify two key frames from each video: (i) the apex frame, representing the peak of the happiness expression, and (ii) a neutral frame, typically the initial frame, as participants were instructed to transition from a neutral to a happiness expression and return to neutral during the recording (12). Happy expression was selected as the target emotion because it generates consistent motor patterns through smiling, constitutes one of the most prominently affected facial expressions in PD, and engages balanced activation of both upper and lower facial muscle groups.

All automatically selected apex frames were subsequently reviewed manually by an experienced rater. If the frame did not visually correspond to the point of maximal expression, it was replaced with the correct frame, chosen within a ±20-frame window around the detected apex. This review step was applied to every video to ensure that all apex frames used in the analysis were validated as correct before feature extraction. From these selected frames, the coordinates of 68 facial landmarks were extracted using the dlib facial recognition library (Supplementary Figure S1) (13). Using these landmark coordinates, a total of 30 Euclidean distances were computed across three facial regions: 10 from the lower face, 10 from the upper face, and 10 from a combination of both. The combined distances were carefully selected to capture the largest magnitude of displacement while minimizing the feature space dimensionality, which helps reduce overfitting and improves model generalizability (Supplementary Table S1). The Euclidean distance between two landmarks, with coordinates ( $x_{1}$ , $y_{1}$ ) and ( $x_{2}$ , $y_{2}$ ), was calculated using the standard formula;


$$d=\sqrt{{\left(x_{2}-x_{1}\right)}^{2}+{\left(y_{2}-y_{1}\right)}^{2}}$$

These features were computed for both the neutral and apex frames of the happiness expression, yielding a total of 20 features for each of the three defined facial regions. A detailed list of all features is presented in Supplementary Table S1. Notably, the selected features were informed by a thorough review of prior literature on hypomimia and facial image analysis (14–17), ensuring their relevance and validity for assessing facial expressivity in PD.

Next, correlations between the 20 extracted facial features and the ROMP-saliva scores were assessed through a three-step analytical process. First, principal component analysis (PCA) was performed to reduce the dimensionality of the feature space, retaining the components that captured the most variance in the data while minimizing redundancy and enhancing computational efficiency. Second, canonical correlation analysis (CCA) was applied to evaluate the relationships between the resulting principal components (PCs) and the nine individual items of the ROMP-saliva scale (18) enabling the identification of multivariate associations between facial features and drooling severity. Finally, bootstrapping was employed to assess the stability and significance of the CCA-derived correlation (19). This analytical sequence was conducted separately for each of the three facial regions (upper, lower, and combined).

Pearson’s correlation coefficient was used to rate the correlation between the severity of hypomimia and drooling. For group comparison for the hypomimia score between the drooling and non-drooling group, independent sample *t*-test was used after Shapiro–Wilk test for normality assessment. The data was analyzed using IBM SPSS version 29.0.1.0 for Windows, and Python. *p* values of < 0.05 were considered statistically significant. Correlations were considered strong if *r* > 0.7, moderate if 0.5 ≤ *r* ≤ 0.7, and weak if *r* < 0.5.

## 3 Results

### 3.1 Association between hypomimia and drooling scales

**Figure 1:**
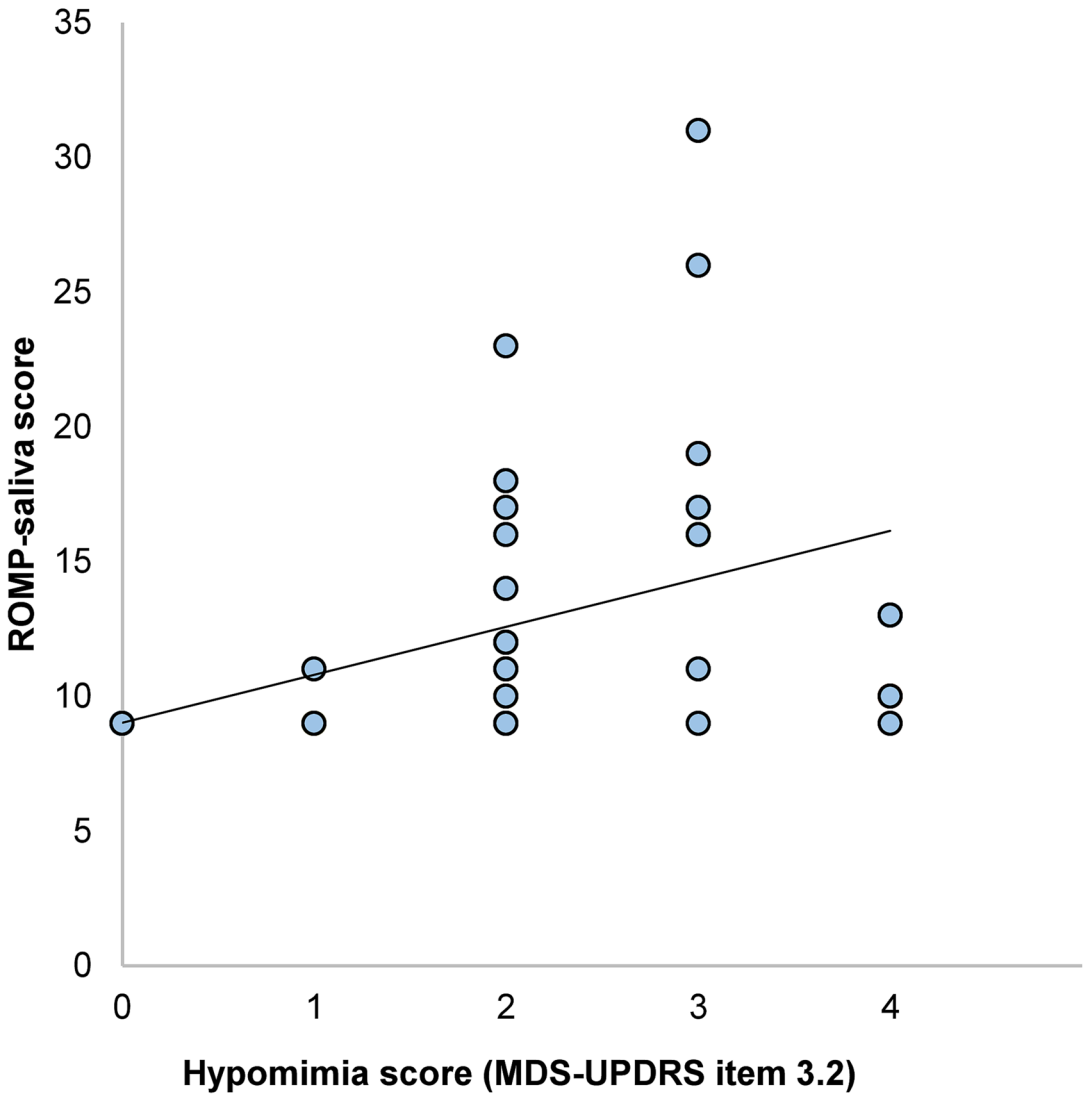
Scatterplot showing the relationship between hypomimia and drooling severity scores with correlation line.

A total of 104 patients met the study’s inclusion criteria. Of these, 52 participants who completed both the ROMP-saliva questionnaire and facial video recordings were included in the final analysis. Of note, the high exclusion rate (50%) was due to our study design where facial expression recording was optional within a broader motor assessment protocol. The mean ROMP-saliva score, reflecting drooling severity, was 12.8 ± 4.8, while the mean MDS-UPDRS item 3.2 score, assessing hypomimia, was 2.1 ± 0.1 (Table 1). Pearson correlation analysis revealed a weak but statistically significant positive correlation between hypomimia and drooling severity (*r* = 0.368, *p* = 0.007; Figure 1).

**Table 1:** Baseline demographics of the study.

Characteristics	*n* = 52
Age, years	67 ± 9.2
Sex, male (%)	31 (60%)
ROMP-saliva score	12.8 ± 4.8
MDS-UPDRS 3.2 item score	
0	3 (6%)
1	8 (15%)
2	25 (48%)
3	11 (21%)
4	5 (10%)

When stratified by drooling status, defined by a ROMP-saliva score >9 (drooling group, *n* = 34) versus ≤9 (non-drooling group, *n* = 18), participants in the drooling group demonstrated significantly higher hypomimia scores (2.5 ± 0.7) compared to the non-drooling group (1.4 ± 1.0; *p* < 0.001).

### 3.2 Correlation assessments through facial video analyses

Principal component analysis (PCA) was performed with a 95% variance retention threshold, resulting in dimensionality reduction of the original 20 facial features to 10 principal components (PCs) for both the lower and upper facial regions, and 8 PCs for the combined region (Supplementary Figure S2). These PCs were subsequently analyzed using canonical correlation analysis (CCA) in conjunction with the nine individual items of the ROMP-saliva questionnaire (Table 2). This analysis yielded five canonical correlation (CC) pairs linking the facial PCs (X CCs) with the ROMP-saliva items (Y CCs).

**Table 2:** Nine individual subitems of the ROMP-saliva scale used in the CCA.

Subitem	Question
1	Do you experience loss of saliva during the day?
2	How often do you experience increased amount of loss of saliva?
3	Do you experience loss of saliva during the night?
4	Does your (loss of) saliva impair your eating and drinking?
5	Does your (loss of) saliva impair your speech?
6	What do you have to do to remove saliva?
7	Does the loss of saliva limit you in contacts with others?
8	Does your loss of saliva limit you in doing activities inside or outside your home (work, hobbies)?
9	How bothered are you as a result of your (loss of) saliva?

Following bootstrapping, the correlations between the X and Y canonical components were computed. Statistically significant correlations were observed between the X and Y canonical components across all facial regions (Table 3). For all regions, correlations were highest for CC1 (*r* = 0.799 for lower and upper regions; *r* = 0.722 for combined regions, all *p* < 0.001) and decreased for subsequent components. Mean correlation coefficients were highest for the upper facial region (0.641), followed by the lower (0.600) and combined (0.575) regions.

**Table 3:** Correlations between X and Y canonical components (CCs) for lower, upper, and combined facial regions following bootstrapping.

CC	Lower facial region (p)	Upper facial region (p)	Combined facial region (p)
1	0.799 (<0.001)	0.799 (<0.001)	0.722 (<0.001)
2	0.699 (<0.001)	0.719 (<0.001)	0.665 (<0.001)
3	0.567 (<0.001)	0.653 (<0.001)	0.626 (<0.001)
4	0.497 (<0.001)	0.580 (<0.001)	0.472 (<0.001)
5	0.414 (0.002)	0.456 (<0.001)	0.392 (0.004)
Mean	0.600	0.641	0.575

## 4 Discussion

This study investigated the association between hypomimia and sialorrhea in patients with PD using an integrated approach combining quantitative facial expression analysis and a validated clinical drooling scale. By applying PCA and CCA to the extracted facial features, we identified moderate canonical correlations between ROMP-saliva scores and both lower facial (mean *r* = 0.600) and upper facial (mean *r* = 0.641) features. The CCA revealed five statistically significant canonical correlations across the lower, upper, and combined facial regions. For the lower face, coefficients ranged from 0.799 (*p* < 0.001) for the first dimension to 0.414 (*p* = 0.002) for the fifth, possibly reflecting hypomimia-related restrictions in lower-face expressivity and diminished oral motor control, both associated with sialorrhea severity. The upper face showed a comparable CC1 (0.799, *p* < 0.001) but a slightly higher mean correlation, suggesting a more distributed variance across dimensions, potentially linked to hypomimia-related brow and eye aperture changes. The combined facial region yielded CC1 = 0.722 (*p* < 0.001) but a lower mean (0.575), indicating that mixing upper- and lower-face variables may dilute region-specific effects. Lower-order canonical correlations (CC4–CC5) explained minimal additional variance, likely reflecting subtle or non-specific associations. Overall, these results support the potential utility of facial motion patterns as surrogate markers for the screening of sialorrhea in clinical PD settings.

Our findings revealed a significant positive correlation between hypomimia and drooling severity in PD, consistent with prior observational studies that have suggested a link between these motor and non-motor manifestations. These findings build upon earlier observations linking sialorrhea to impaired orofacial motor control (4), and suggest potential relationship between facial expressivity and drooling in patients with PD (8). Previous research has employed various assessment tools to quantify these symptoms, including the drooling item from the UPDRS Part II, the Drooling Severity and Frequency Scale, and the facial expression subscore from MDS-UPDRS Part III (20, 21). It is noteworthy that the ROMP questionnaire is currently the only scale endorsed by the Movement Disorder Society for evaluating drooling in PD (22). A prior study using the ROMP-saliva scale was limited to group comparisons based on predefined UPDRS hypomimia cutoffs (22). In contrast, our study employed both continuous clinical scores and AI-derived video features to provide a more granular analysis of the hypomimia–sialorrhea relationship, thereby enhancing the robustness and resolution of our findings.

Interestingly, we observed that participants with the most severe hypomimia (MDS-UPDRS item 3.2 score = 4) reported lower ROMP-saliva scores (Figure 1). This finding suggests a potential nonlinear relationship between hypomimia severity and reported drooling symptoms. Several explanations may account for this finding. Patients with more advanced motor impairment may be less aware of or less likely to report drooling, potentially due to reduced insight or concurrent cognitive decline. Alternatively, patients with the most severe drooling may already be under treatment for drooling symptoms. Finally, the relatively small sample size (*n* = 52) of the study may have limited the reliability of this pattern. Whether this inverted U-shaped relationship remains consistent warrants validation in larger cohorts.

Application of PCA and CCA revealed that facial features from the upper, lower, and combined facial regions were moderately associated with drooling severity. Notably, the strongest correlations were observed in periocular features (*r* = 0.641), exceeding those from perioral features (*r* = 0.600). This was somewhat unexpected given the presumed pathophysiological link between reduced perioral motor control and impaired saliva clearance. Sialorrhea in PD is believed to arise from multiple contributing factors, including increased saliva production, impaired oral retention, and diminished clearance due to orofacial bradykinesia (4). Some studies have also implicated cognitive impairment in the exacerbation of drooling severity (23). Our findings suggest that while perioral dysfunction remains important, broader facial motor impairment—potentially reflecting more widespread basal ganglia circuit disruption—may play a more central role in the pathophysiology of drooling in PD.

Drooling remains a common but under-recognized symptom in PD, despite its well-documented impact on quality of life and the availability of effective treatments, including speech-language therapy, botulinum toxin injections, and pharmacologic interventions (6). Our study highlights the potential of quantitative facial analysis with the aid of AI to serve as a non-invasive, automated tool for predicting sialorrhea, thereby improving early detection and clinical monitoring. Recent advances in digital biomarkers for PD, such as wearable sensors and vision-based assessments, have shown promise for evaluating motor features (9, 24). Video-based hypomimia assessment tools, which are already under active development (14, 17), may also be extended to screen for non-motor symptoms like drooling—broadening the scope of digital phenotyping in PD.

This study has several limitations. First, we did not collect detailed demographic or clinical information such as disease duration, medication status, or total MDS-UPDRS Part III scores. This was an intentional decision aimed at simplifying data collection and promoting remote, patient-friendly assessment, aligning with the broader goals of digital healthcare. Second, the study was limited to evaluating associations and did not develop predictive models for clinical deployment. Future work should focus on building AI-based systems that can estimate drooling severity and support therapeutic decision-making, such as determining the need for botulinum toxin treatment or referral to speech therapy. Third, our analysis was based on static apex–neutral frame comparisons and did not capture dynamic features such as tremor or freezing episodes. Incorporating time-series or frequency-based features would require a larger dataset to ensure robust modeling. In addition, while we employed linear methods (PCA and CCA) to ensure interpretability and robustness, we acknowledge that some facial abnormalities in PD may follow nonlinear patterns. Future work employing nonlinear approaches, such as neural networks, may better capture these complex associations, particularly with larger datasets. Fourth, we focused exclusively on happiness as the main target emotion, which may not fully represent the range of facial motor dysfunction in PD. Different emotions engage distinct muscle groups, and incorporating multiple expressions could provide a more comprehensive assessment of facial motor function. Lastly, our analysis was cross-sectional and does not provide insight into the causal or mechanistic links between hypomimia and sialorrhea. Longitudinal studies incorporating multimodal data—including neuroimaging, autonomic testing, and cognitive assessments—will be necessary to elucidate the underlying pathophysiological mechanisms and validate the predictive potential of facial features in PD.

## 5 Conclusion

This study provides novel evidence supporting an association between hypomimia and sialorrhea in PD and demonstrates the feasibility of using AI-based video analysis to evaluate these features objectively. By identifying facial motion characteristics as potential digital biomarkers for drooling, our findings lay the groundwork for more accessible, scalable, and non-invasive assessment strategies for this often-overlooked non-motor symptom. Such approaches may ultimately enhance early detection and enable more timely, targeted interventions, thereby contributing to improved quality of life for individuals living with PD.

## Data Availability

The datasets presented in this article are not readily available because they include facial information of the participants. Requests to access the datasets should be directed to Juana A. Castaneda, juana.ayala@ubc.ca.
